# Laparoscopic Cholecystectomy in Moynihan's Hump, Varices of Paracholedochal Plexuses With Superficial Course of Inferior Vena Cava at Calot's Triangle: Case Report

**DOI:** 10.1002/ccr3.70593

**Published:** 2025-07-01

**Authors:** Imtiaz Wani

**Affiliations:** ^1^ Department of Minimal Access and General Surgery Government Gousia Hospital, DHS Srinagar Kashmir India

**Keywords:** congenital anomalies, inferior vena cava, Moynihan's hump, paracholedochal plexuses, portal vein, safe cholecystectomy, varices

## Abstract

Anatomical variations of the vascular supply of the hepatobiliary system are rare to see. A sound knowledge of congenital variations of vascular patterns in and around Calot's triangle is essential for the safe practice of laparoscopic cholecystectomy. A case of 34‐year‐old female is reported who has intraoperative diagnosis of Moynihan's hump, varices of paracholedochal plexuses, superficially placed portal vein in anterior plane with superficial course of inferior vena cava at Calot's triangle during laparoscopic cholecystectomy. An uneventful laparoscopic cholecystectomy was done with utmost meticulous approach and careful surgical dissection in Calot's triangle to avoid any inadvertent injury to anomalous vasculature. An occurrence of congenital anomaly of Moynihan's hump, varices of paracholedochal plexuses, superficially placed portal vein in anterior plane with superficial course of inferior vena cava at Calot's triangle is extremely rare and incidentally diagnosed intraoperatively. This case report is the first kind of case of world reporting occurrence of a multiple vascular anomalies diagnosed during laparoscopic cholecystectomy. Culture of safe practice of laparoscopic cholecystectomy is to be advocated and this is to be called as “Srinagar Cholecystectomy”.

## Graphical abstract

During laparoscopic cholecystectomy, intraoperative findings of varices of paracholedochal plexuses, superficially placed portal vein and Moynihan's hump observed. The common bile duct placed on the inferior vena cava which was superficially placed at Calot's triangle. Srinagar Cholecystectomy is to be always advocated in Simple to dangerous cholecystectomy.

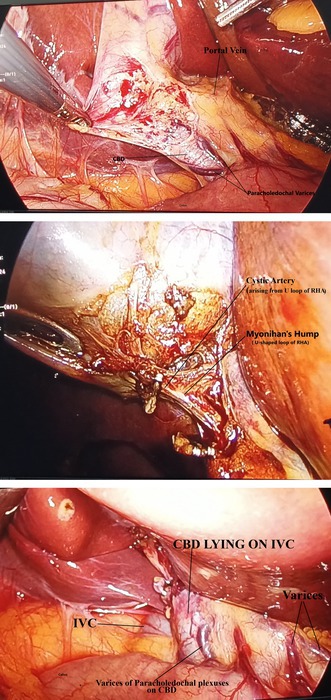

**Summary**

- An occurrence of congenital vascular anomaly of Moynihan's hump, varices of paracholedochal plexuses, superficially placed portal vein in anterior plane with superficial position of IVC at Calot's triangle is an extremely rare occurrence during laparoscopic cholecystectomy. Preoperative diagnosis is difficult. Culture of safe practice of laparoscopic cholecystectomy or Srinagar cholecystectomy is to be advocated.

## 1 Introduction

The concept of a culture of safe cholecystectomy is being ubiquitously advocated for the performance of laparoscopic cholecystectomy [1]. In the presence of congenital vascular anomalies, laparoscopic cholecystectomy is always challenging and of utmost clinical significance. If unrecognized, any inadvertent injury to these congenital anomalous vasculature during laparoscopic cholecystectomy is fraught with disastrous consequences and mortality. These vascular anomalies have been reported to have an incidence rate of conversion to open surgery of approximately 0%–1.9%, with a mortality rate of approximately 0.02% [2].

The incidence of Moynihan's hump, also called Caterpillar hump, of the right hepatic artery ranges between 3% and 13.3% [3]. Correct identification of this caterpillar hump is essential as the cystic artery arising from the loop is typically short, prone to easily avulse from the hepatic artery during laparoscopic cholecystectomy [4, 5]. Varices of bile duct plexuses are always at risk for bleed or rupture during lap cholecystectomy. Portal vein as well as Inferior Vena Cava injuries during laparoscopic cholecystectomy have been reported in the literature. Identification and maintaining the integrity of Moynihan's hump and varices of paracholedochal plexuses in the presence of a superficially placed portal vein with superficial location of IVC around the hepatocystic triangle for achieving a critical view of safety is to avoid iatrogenic vascular injuries with torrential hemorrhage. Managing these vascular injuries is always confronted with high morbidity and mortality. This work was reported in line with the SCARE criteria [6].

## 2 Case History/Examination

A case of a 35‐year‐old female complained of recurrent colicky abdominal pain in the right upper abdomen for 2 months. There was a history of dyspepsia. No history of jaundice, fever, or recent acute attack of cholecystitis was present. General examination was unremarkable and systemic examination did not reveal any significant findings. Per abdominal examination was suggestive of a soft and non‐tender abdomen, except for an LSCS scar seen. Laboratory values were within normal ranges.

## 3 Methods

**FIGURE 1:**
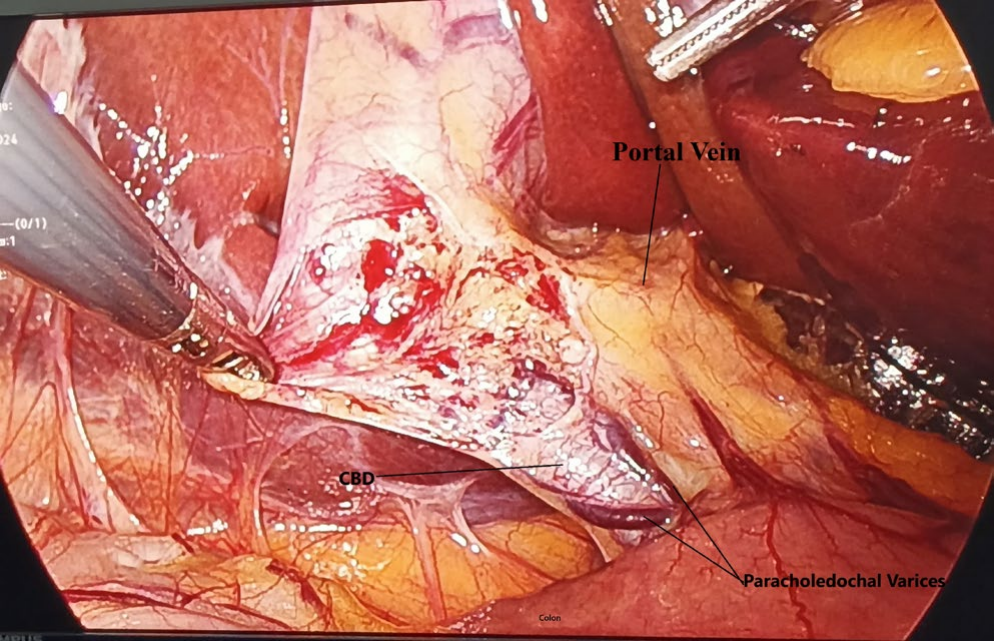
Showing paracholedochal plexus present on common bile duct with anteriorly placed Portal Vein lateral to CBD and hepatic artery.

**FIGURE 2:**
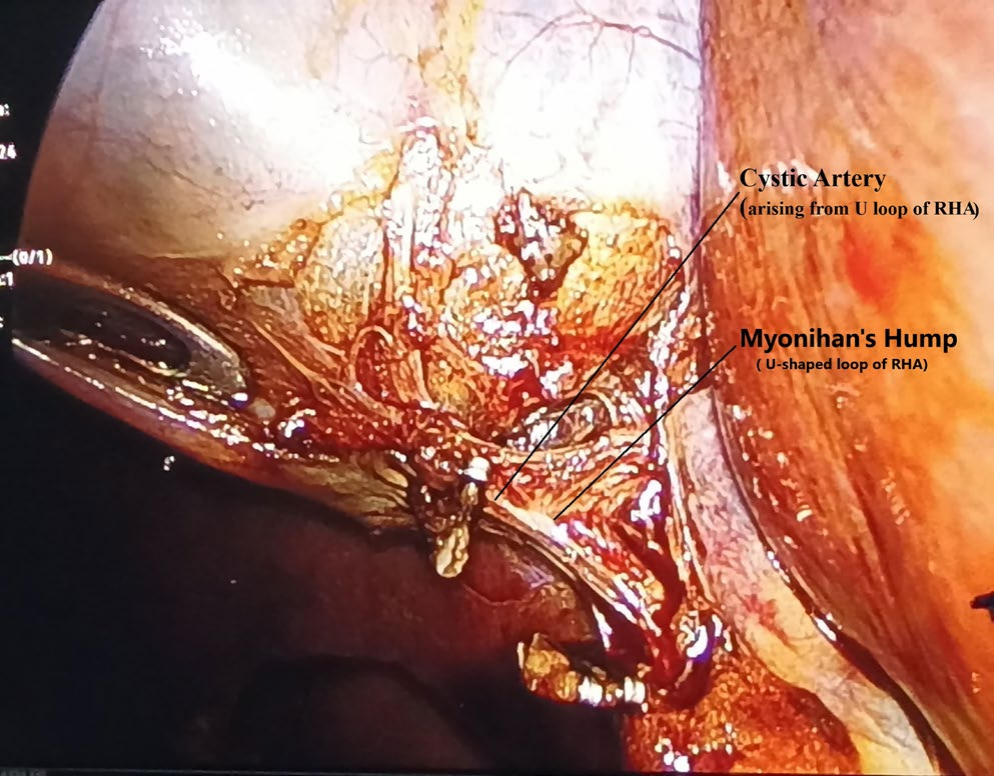
Showing U‐shaped loop of Right Hepatic artery with short cystic artery arising from Moynihan's hump.

**FIGURE 3:**
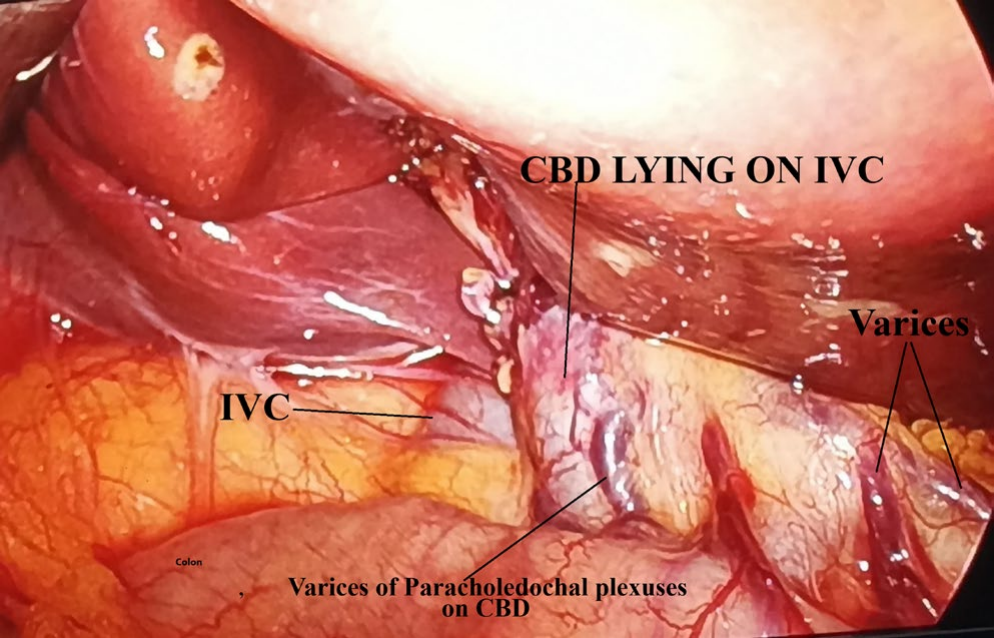
Showing CBD placed on IVC in Calot's triangle with Varices seen.

Upright X‐ray abdomen was normal. ECG was of normal sinus rhythm. On ultrasonography of the abdomen, she had a diagnosis of grade I fatty liver and cholelithiasis. Gallbladder was having multiple calculi inside, wall thickness being 2 mm and with no pericholecystic fluid seen. Rest of the study on ultrasonography was unremarkable. The patient was planned for elective laparoscopic cholecystectomy. She had general anesthesia and four‐port cholecystectomy was done. Intraoperative findings revealed flimsy adhesions between the gallbladder and omentum. Common bile duct was normal in diameter and position. There were multiple engorged venous plexuses present on the common bile duct suggestive of the varices of paracholedochal plexuses of the bile duct (Figure 1) A thin short cystic artery originating from the convexity of a single U‐shaped loop of the right hepatic artery confirmed the occurrence of Moynihan's hump (Figure 2). After further stripping of adhesions from the gallbladder with dissection for the critical view of safety, the inferior vena cava was found pursuing a short superficial course just before entering the groove of the liver at Calot's triangle with CBD placed on IVC (Figure 3). There were a few multiple dilated venous plexuses present, and the portal vein was superficially anteriorly placed lateral to the common bile duct and hepatic artery in the porta hepatis. The portal vein was coursing posterior to the first part of the duodenum but lying in superficial anterior planes. An extremely cautious, patient, and meticulous surgical approach for the critical view of safety was executed. She had an uneventful laparoscopic cholecystectomy with the postoperative period being uneventful. Follow‐up period was uneventful and discharged with proper medical advice. Histopathology of the gallbladder specimen was suggestive of chronic cholecystitis.

## 4 Conclusion and Results

Occurrence of Moynihan's hump, varices of paracholedochal plexuses, superficially placed portal vein in the anterior plane with a superficial course of IVC at Calot's or hepatocystic triangle is of an extremely rare occurrence. These congenital anomalies are rarely diagnosed preoperatively and are often found incidentally during laparoscopic cholecystectomy. An iatrogenic injury to any of these quadruple vascular anomalies is associated with high morbidity and mortality. An extremely meticulous and diligent dissection in Calot's triangle is required for achieving a critical view of safety for laparoscopic cholecystectomy in such a conglomeration of vascular anomalies. In the presence of a number of vascular anomalies, this made laparoscopic cholecystectomy a dangerous procedure to be performed, though it was done successfully. Srinagar Cholecystectomy or Safe Cholecystectomy is to be practiced and advocated.

## 5 Discussion

Bleeding complications due to vascular injuries represent an important cause of morbidity and mortality during laparoscopy, as bleeding control could be technically challenging in inexperienced hands [2]. Congenital vascular anomaly of the hepatobiliary system is a relatively uncommon, and there are a myriad of vascular anomalies if present. Moynihan's hump is characterized by the right hepatic artery coursing in a *U*‐shaped loop very close to the gallbladder as well as the cystic duct forming a short cystic artery [7]. There are multiple etiologies proposed, but none of these have been documented to suggest near causation of the development of Moynihan's hump. Partial or complete persistence of the arterial supply of the fetal liver could lead to the caterpillar hump of the right hepatic artery [3]. As per Benson and Page [8], this is not an anomaly but an artifact formed due to traction during cholecystectomy. Taylor et al. [9] suggested architectural distortion in cirrhosis of the liver causing corkscrewing of intrahepatic arteries leading to a tortuous hepatic artery and subsequent Moynihan's hump.

On an anatomical point of view, this tortuous part of the right hepatic artery pursues a course anterior to or posterior to the common hepatic duct. The posterior location is relatively more common (60%) than the anterior position [10]. In our case, the artery was positioned anterior to the hepatic duct. Depending on its tortuosity, the artery can course with a single loop (55% cases) when it is less tortuous and in a double loop when it is more tortuous. The cystic artery is usually short whether it arises from a single loop or a double loop course, except when it arises from the proximal loop of the right hepatic artery [11]. This anomaly is prone to lead to complications from the misidentification of the right hepatic artery as a cystic artery, tractional avulsion injury, and direct injury to the right hepatic artery or cystic artery during dissection for achieving the critical view of safety. Any inadvertent injury to Moynihan's hump often leads to torrential bleeding, obscuring the view for achieving the critical view of safety and posing a risk for bile duct injury in an attempt at blind salvage for control of bleeding. These unforeseen intraoperative complications of uncontrolled bleeding from the Caterpillar hump prompt conversion from laparoscopic to open cholecystectomy [12]. Uncontrollable bleeding or misinterpretation of the hepatic artery as the cystic artery often leads to clipping of the right hepatic artery. If there is incomplete clipping, a pseudoaneurysm of the hepatic artery may form. Accidental complete clipping of the right hepatic artery may cause right lobar ischemic necrosis of the liver. This congenital Moynihan's hump may rarely coexist with other anomalous vasculature and ducts of the hepatobiliary system.

Types of Moynihan's hump are to be classified as per “Srinagar Classification” of Moynihan's hump as:

Type I: Isolated Moynihan's hump of any type present.

Type II: Moynihan's hump associated with other types of vascular anomaly of hepatobiliary system.

Type III: Moynihan's hump associated with anomaly of ducts of hepatobiliary system.

Type IV: Moynihan's hump associated with both anomaly of vascular supply and ducts of hepatobiliary system.

The extrahepatic bile duct is surrounded by two venous systems, namely the paracholedochal veins of Petren and the epicholedochal venous plexus of Saint [13]. The paracholedochal veins are extrinsic to the bile duct wall, and their varices may cause extrinsic compression. The Saint plexus, on the contrary, is a fine reticular venous network intimately covering the outer surface of the common hepatic and bile duct. Varices of epicholedochal plexuses may present as upper gastrointestinal bleed. These variceal plexuses in laparoscopic cholecystectomy have been impounded as high‐risk cases for torrential bleeding in the event of an accidental injury to these. Varices of paracholedochal plexuses are usually offered treatment when complications develop. In the presence of varices of portal plexuses in the hepatocystic triangle, laparoscopic cholecystectomy is considered to be a high risk [14]. There are very few reports of isolated portal vein injury without associated biliary lesions [2]. A superficially placed portal vein with gross visibility on anterior planes passing posterior to the duodenum was lateral to the CBD and hepatic artery, posing minimal risk of injury during dissection in Calot's triangle for achieving the critical view of safety.

Injuries to IVC are very rare and are considered a life‐threatening complication of laparoscopic cholecystectomy [15]. Such inferior vena cava injury is frequently associated with trocar or Veress needle insertion during laparoscopic cholecystectomy. Early and prompt diagnosis of IVC injury, with a priority call for a vascular expert, is mandatory for the management of this serious injury to minimize morbidity and mortality. Very rarely, injury to the right renal artery leading to the formation of a pseudoaneurysm with a subsequent renal–vena cava fistula has been reported [16]. This is usually a late complication following laparoscopic cholecystectomy. The risk of venous air embolism has been associated with inferior vena cava injury in laparoscopic cholecystectomy, with severity ranging from being asymptomatic to cardiovascular collapse [17, 18].

There is a possibility of disastrous sequences of events in laparoscopic cholecystectomy for any inadvertent injury to varices of paracholedochal plexuses, of aberrant superficial position of IVC, an accidental avulsion, or misidentification of the right hepatic artery as a cystic artery from Moynihan's hump. A thorough search of PubMed, Embase, Web of Science, Google for literature review on dangerous laparoscopic cholecystectomies performed authenticates that this case is among one of the most dangerous laparoscopic cholecystectomies done for symptomatic cholelithiasis in the presence of quadruple vascular anomalies in the world from the inception of laparoscopic cholecystectomy till date. Adequate surgical experience, astounding knowledge of congenital hepatobiliary vascular anomalies, egregious caution, and diligent meticulous dissection for critical view of safety during laparoscopic cholecystectomy is crux for avoidance of any vascular injury.

## Author Contributions

**Imtiaz Wani:** conceptualization, investigation, writing – original draft, writing – review and editing.

## Disclosure

The author has nothing to report.

## Consent

Written informed consent was obtained from the patient to publish this report in compliance with the journal's patient consent policy.

## Conflicts of Interest

The author declares no conflicts of interest.

## Data Availability

Due to the protection of the privacy rights of patient, the data is not published publicly. Contact the corresponding author's email address more clinical data is available on request.
